# Management of a Displaced, Osteoporotic Cuboid Fracture: A Case Report

**DOI:** 10.7759/cureus.91186

**Published:** 2025-08-28

**Authors:** Claire Cotton, Ryan Langston, Joshua Fernicola, Jake Knight, Jason Bowman

**Affiliations:** 1 Orthopaedic Surgery, Medical College of Georgia at Augusta University, Augusta, USA; 2 Orthopaedic Surgery, Southeast Georgia Health System, Brunswick, USA

**Keywords:** bone healing, cancellous allograft, comminuted, cuboid, cuboid dislocation, cuboid fracture, demineralized bone matrix, lateral column, open reduction internal fixation, osteoporosis

## Abstract

Cuboid fractures are relatively rare injuries to the midfoot that commonly occur simultaneously with additional fractures and disruptions to different articular surfaces. Due to the cuboid’s large articular surface and contribution to the maintenance of lateral column stability, treatment of cuboid fractures can be difficult and manifest with a variety of post-operative complications. Although simple cuboid fractures can be managed conservatively, at this time, there is no standard operative approach for displaced cuboid fractures. Furthermore, the management of these fractures can be further complicated by poor bone quality, making them unamenable to traditional open reduction and internal fixation (ORIF) and plate fixation. This case report outlines the diagnosis and surgical management of a closed cuboid fracture-dislocation in a 43-year-old female patient with osteoporosis.

## Introduction

Cuboid fractures are uncommon yet serious injuries that can lead to chronic pain and disability [1-3]. Isolated cuboid fractures are rare, and injury to the cuboid is often associated with additional fractures and soft tissue injury, especially to the Lisfranc and Chopart complexes [4,5]. Cuboid fractures most commonly present as avulsion injuries, though compression fractures are also observed [6]. The most well-documented compression fracture of the cuboid is the nutcracker fracture, which is believed to result from longitudinal compression of the lateral column of the foot due to axial loading of the heel while the foot is in a plantarflexed and abducted, fixed position [7,8]. Comminuted fractures occur due to direct impact on the lateral aspect of the foot. Long-term complications include residual articular incongruity of the calcaneocuboid and fourth and fifth tarsometatarsal (TMT) joints, malunion of the body of the cuboid and lateral column, shortening resulting in forefoot abduction with lateral subluxation of the lesser metatarsals, and residual planus or planovalgus deformity [9,10]. No long-term outcome studies with validated objective scoring systems have been published. Outcomes are confounded by simultaneous injuries to other structures [1].

Because of its shape and ligament attachments, fractures of the cuboid are usually associated with other midfoot injuries. The cuboid is involved in all the intrinsic movements of the midfoot and hindfoot [11]. While conservative management is acceptable for some cuboid fractures, surgical intervention is recommended for injuries with displacement of articular surfaces and or shortening of the lateral arch of the foot [2,11,12]. Nondisplaced intraarticular and extraarticular cuboid fractures can be managed conservatively with 4 to 6 weeks of non-weight bearing in a below-the-knee cast or pneumatic boot and serial radiographs to assess for any displacement, shortening, or avascular necrosis [2].

Osteoporotic fractures are more susceptible to non-union due to diminished bone quality, mechanical instability, and impaired bone regeneration, which involves reduced osteoblast function and poor vascularity. Standard treatment strategies include medical therapy, such as bisphosphonates and teriparatides, with potential surgical management involving mechanical fixation and bone grafting [13]. Here we describe a case of a comminuted cuboid fracture complicated by additional midfoot injuries in a 43-year-old female patient with a history of osteoporosis secondary to premature ovarian failure and primary hyperparathyroidism.

## Case presentation

**Figure 1:**
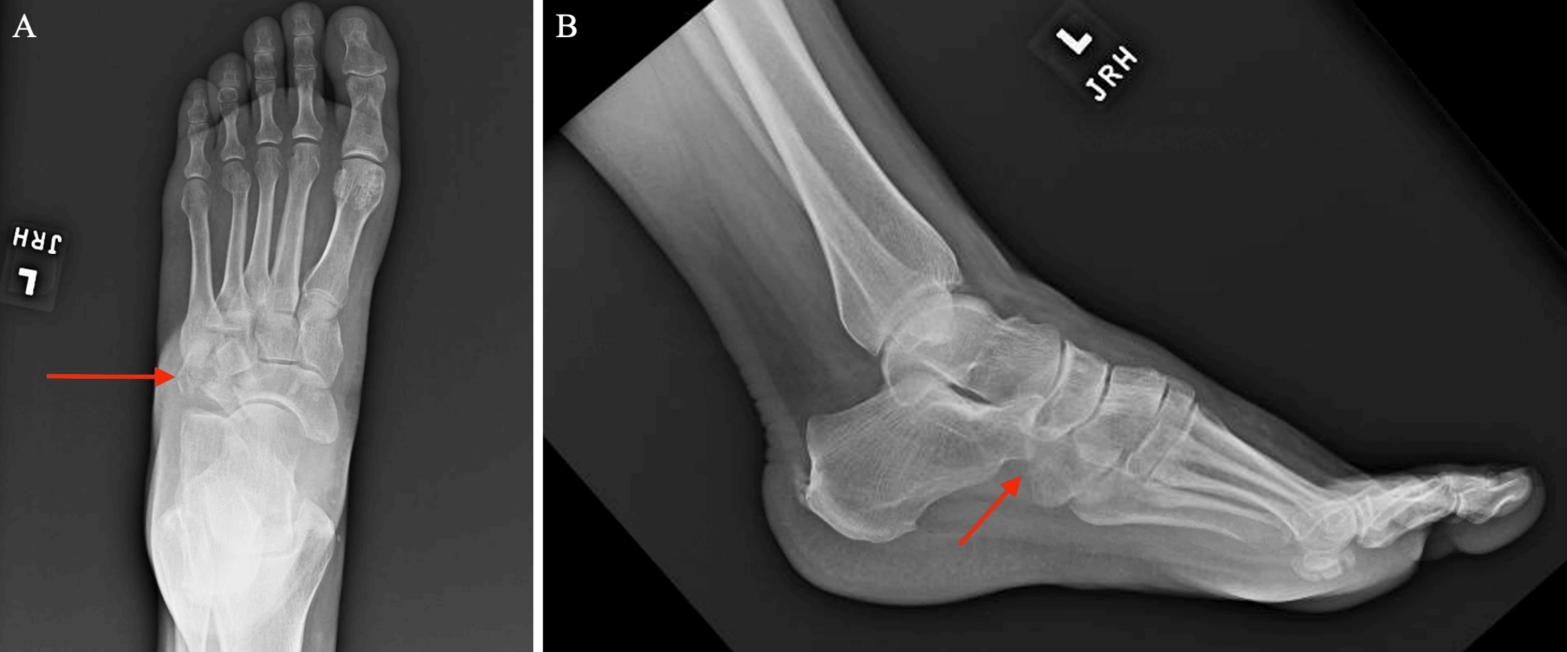
Initial anteroposterior and lateral X-ray of the left foot with a comminuted cuboid fracture (red arrow) A: Anteroposterior view. B: Lateral view.

**Figure 2:**
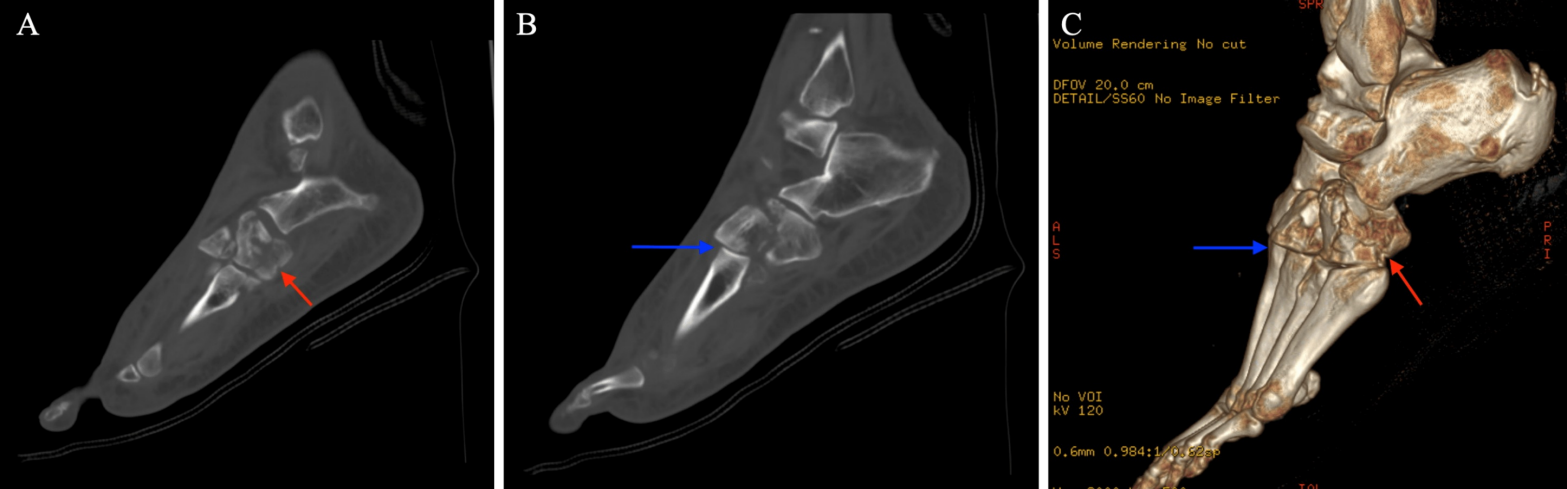
Initial sagittal CT and 3D volume rendering technique CT of the left foot with a comminuted cuboid fracture (red arrow) and third TMT joint dislocation (blue arrow) A: Sagittal CT at the level of the cuboid. B: Sagittal CT at the level of the third tarsometatarsal joint. C: 3D volume rendering technique CT with a comminuted cuboid fracture and third TMT joint dislocation. CT: computed tomography. 3D: three-dimensional. TMT: tarsometatarsal.

A 43-year-old woman, with a history of diagnosed osteoporosis on a dual-energy X-ray absorptiometry (DEXA) scan secondary to premature ovarian failure and primary hyperparathyroidism, presented to the clinic with pain and swelling of the lateral aspect of her left midfoot. The injury occurred when the patient fell through a weak spot in her wooden floor. Plain films (Figure 1) and CT imaging (Figure 2) revealed a comminuted cuboid fracture with shortening of the lateral column, a fracture dislocation of the lateral cuneiform with dislocation of the third TMT, and non-displaced fractures of the medial and middle cuneiforms. This incredibly challenging and uncommon fracture pattern was made even more complex by her osteoporotic bones. Her most recent DEXA from three months prior to injury revealed a T score of -2.5 and a Z score of -2.7 in the left femoral neck. The patient did not have a history of prior osteoporotic fractures. She was not on any anti-osteoporotic agents and had not yet undergone parathyroidectomy at the time of injury. Regardless of the approach to management, the patient is at a very high risk of loss of reduction. 

Surgical risks, benefits, and alternatives were discussed with the patient, who elected to undergo surgery and consented to surgical fixation of the third tarsometatarsal joint (TMTJ) and open reduction and internal fixation (ORIF) of the cuboid with likely cuboid spanning ex-fix or bridge plate. A dorsal incision centered over the second TMTJ was utilized to access the third TMT joint. Sharp dissection was taken through the skin and Bovie electrocautery was used to control hemostasis. Blunt dissection was used for the deep dissection, and the deep neurovascular bundle was identified. These structures were protected and retracted throughout the case. The second and third TMTJ were identified, and the capsule was opened longitudinally. A dorsal incision centered over the third TMTJ was utilized to reduce the third TMTJ. Sharp dissection was taken through the skin and Bovie electrocautery was used to control hemostasis. The third TMTJ was dorsally dislocated. This joint was reduced and pinned with a Kirschner wire. A dorsal plate was then applied as a bridge plate. Intraoperative fluoroscopy was utilized and confirmed satisfactory alignment and position of the hardware. The second TMT joint was exposed and was well reduced and stable. The first TMTJ was stable on X-ray and nontender and stable to the piano key test in the clinic. Intraoperative fluoroscopy was utilized and confirmed satisfactory alignment, bony apposition, and position of the hardware. 

A dorsolateral incision was made over the cuboid leaving adequate skin bridge between this incision and the other incision. We dissected through skin and soft tissue to the level of the cuneiform. This fracture was substantially comminuted with poor-quality bone. This was not surprising given her preoperative imaging and diagnosis of osteoporosis on the DEXA scan. There was a cavern of missing bone in the cuboid. Given this, the cuboid was not amenable to traditional ORIF and plate fixation. The bony defect was grafted with a cancellous allograft and a demineralized bone matrix (DBM) to provide additional bone stock. This was reduced, and then a joint-spanning bridge plate was placed from the anterior process of the calcaneus to the fourth metatarsal base. Fluoroscopy confirmed adequate restoration of the lateral column, safe position of the spanning plate, and adequate restoration of the calcaneocuboid joint and TMT joints, given the poor bone quality and comminuted fracture. 

**Figure 3:**
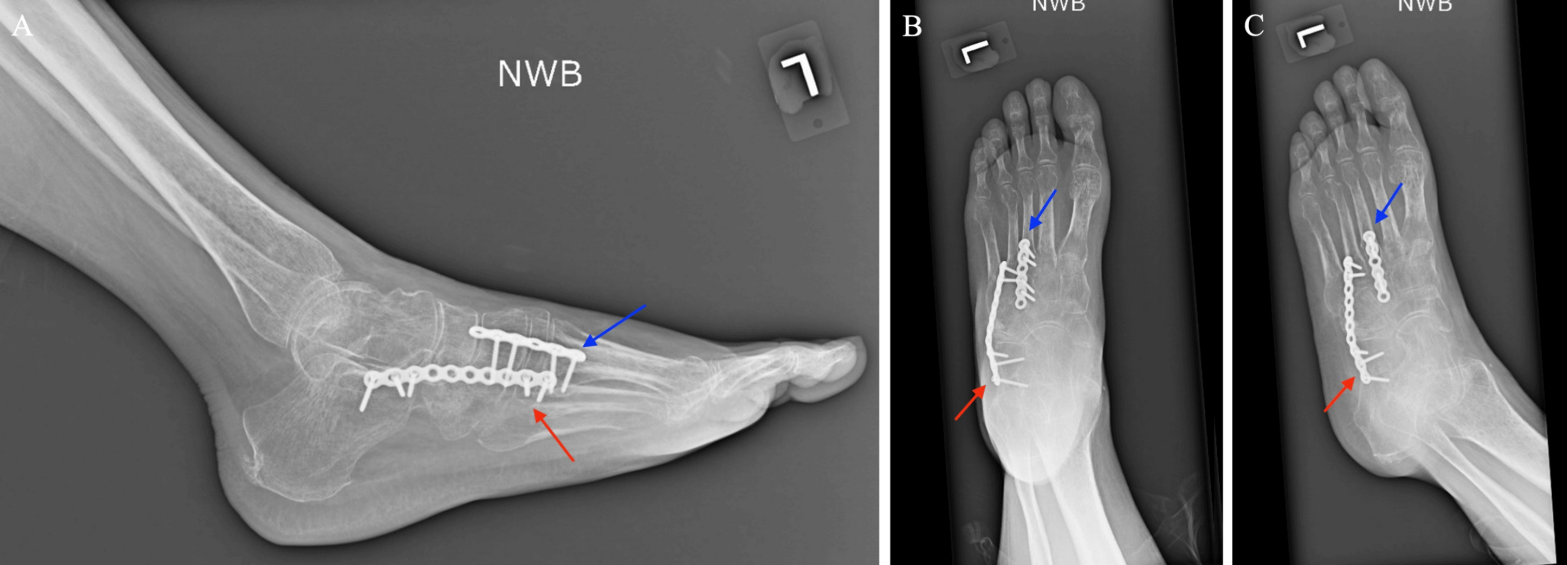
Fourteenth-week post-operative anteroposterior and lateral X-ray of the left foot with a cuboid bridge plate (red arrow) and third TMT joint bridge plate (blue arrow) A: Lateral view. B: Anteroposterior view. C: Lateral oblique view. TMT: tarsometatarsal.

**Figure 4:**
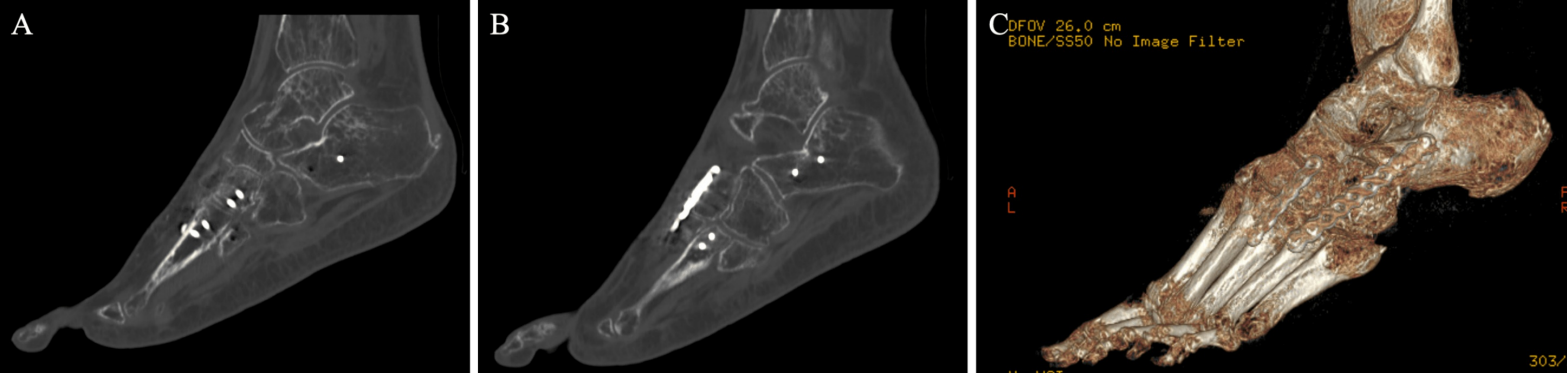
Fourteenth-week post-operative sagittal CT and 3D volume rendering technique CT of the left foot A: Post-operative sagittal CT at the level of the third tarsometatarsal joint. B: Post-operative sagittal CT at the level of the cuboid. C: Post-operative 3D volume rendering technique CT. CT: computed tomography. 3D: three-dimensional.

**Figure 5:**
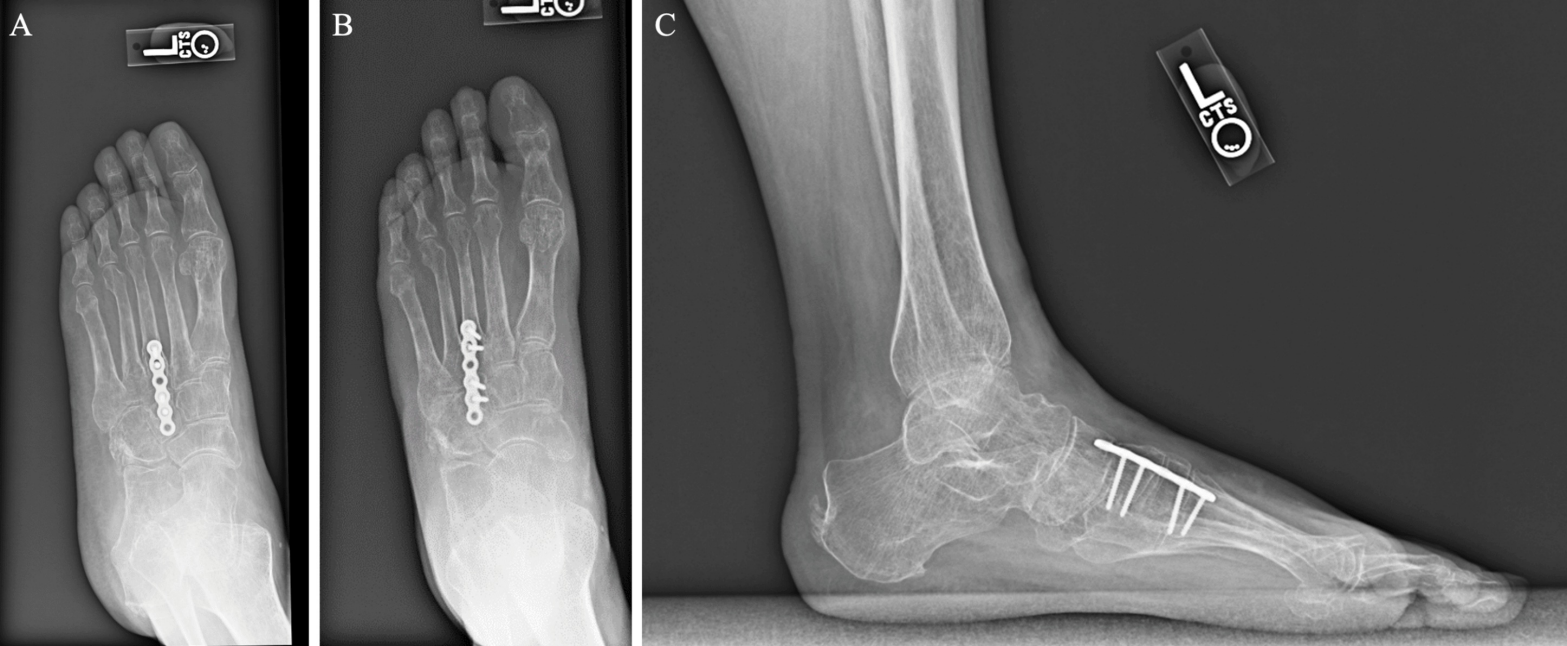
Nineteenth-week post-operative anteroposterior and lateral X-rays after cuboid bridge plate removal with remaining third TMT joint bridge plate in place A: Non-weight-bearing anteroposterior view. B: Weight-bearing anteroposterior view. C: Lateral view. TMT: tarsometatarsal.

Her intraoperative and immediate post-operative courses were uncomplicated. She was followed up at two and a half weeks, four weeks, 10 weeks, 14 weeks, and 19 weeks post-operation, where she reported continued improvement in pain and functionality. Imaging at 14 weeks post-op (Figures 3, 4) revealed stable hardware and united bone. Otherwise, there were no acute fractures of the foot. At four months post-op, the cuboid bridge plate was removed without complications in order to restore motion to the lateral column. Imaging three weeks after cuboid bridge plate removal revealed stable lateral column length without loss of reduction (Figure 5).

A chronic issue for this patient has been the control of her severe osteoporosis. She was followed by an endocrinologist who prescribed calcium and vitamin D supplementation. She also had a parathyroidectomy two months after ORIF with a general surgeon. Three months after removal of hardware, she had returned to her baseline activity level without foot pain. She will continue to follow up with orthopedic surgery at six-month intervals where she will be monitored for potential long-term complications including post-traumatic arthritis of the TMT joints [2]. 

## Discussion

Our literature review failed to identify reports of similar injuries in patients with osteoporosis. Cuboid fractures are rare and are often difficult to effectively treat [14]. In patients without significant co-morbidity, the risk of non-union and malunion in isolated cuboid fractures is rare and has only been documented in the literature as isolated case reports [10]. The patient described in this case represents an exceedingly complex instance of cuboid fracture due to the excessive comminution of her fracture, likely attributable to and exacerbated by her osteoporosis. In addition, there was a substantial cavern of bone missing, which made this patient an unsuitable candidate for traditional ORIF. Osteoporosis is a well-documented cause of non-union and also likely contributed to the poor bone quality [13]. As such, special care was planned to ensure the best-case outcome for this patient's injury. 

This injury was complicated by additional midfoot fractures, which are common in cuboid fractures [6]. The patient underwent surgical fixation of her third TMT joint with a dorsal plate. Additionally, her osteoporosis led to the absence of bone, which required supplemental allograft bone to restore lateral column length. This was achieved with cancellous allograft and demineralized bone matrix. Finally, a joint-spanning bridge plate was placed from the anterior process of the calcaneus to the fourth metatarsal base.

The patient's procedure went well, and no intraoperative complications were noted. In total, the patient underwent ORIF of the left third tarsometatarsal joint and ORIF of the left cuboid with a bridge plate from the anterior process of the calcaneus to the fourth metatarsal base. She was placed into a boot and instructed not to bear weight on the affected limb for eight weeks postoperatively. Follow-up occurred post-operatively at two and a half weeks, four weeks, 10 weeks, 14 weeks, and 19 weeks, and no concerning findings were observed at any of these instances. Approximately four months after ORIF, her lateral column bridge plate was removed. The patient has had an unremarkable recovery period to this point, suggesting a good prognosis for her future. Many patients with similar injuries and treatments will develop some degree of post-traumatic arthritis, which will be a consideration with this patient's treatment moving forward [3].

## Conclusions

This patient's history of severe osteoporosis both increased her chances of cuboid fracture and contributed to the difficulty of the required surgical correction. It was necessary to take special considerations such as incorporating a cancellous bone allograft to further stabilize the fracture and lengthen the lateral column of the foot. Without appropriate management of her osteoporosis, she will be at an increased risk of insufficiency fractures in the future. This case outlines the management and post-operative period of a characteristically poor-healing fracture in a patient with risk factors that also negatively impact her long-term outcome. Her uncomplicated post-operative period supports the success of the treatment strategy used in her case which could be of use to future physicians treating similar cases.
